# Antigenic Stimuli do not Influence Thymic B Lymphocytes: A Morphological and Functional Study in Germ-Free and Conventionally Reared Piglets

**DOI:** 10.1155/1998/57820

**Published:** 1998

**Authors:** B. Cukrowska, I. Trebichavský, P. Rossmann, Z. Řeháková, J. ŠInkora, K. Haverson, R. Lodinová-Žádníková, H. Tlaskalová-Hogenová

**Affiliations:** 1 Department of Immunology and Gnotobiology Institute of Microbiology Academy of Sciences of the Czech Republic Vídeňská 1083 Prague 4 142 20 Czech Republic; 2 Department of Clinical Veterinary Science University of Bristol Langford BS18 7DY UK; 3 lnstitute for Care of Mother and Child Prague Czech Republic

**Keywords:** Thymic B lymphocytes, B-cell differentiation, ELISPOT, pig

## Abstract

We have recently reported that thymic B lymphocytes (TBL) are the first B-cell subpopulation undergoing isotype switching to IgG and IgA during embryonic life. The aim of this study is to analyze the influence of antigenic stimulation on TBL location and activity using a germ-free (GF) newborn pig model, in which maternal antibodies and antigens do not affect B-cell development. Immunohistological analysis showed that TBL were disseminated mainly in the thymic medulla. There were no differences in the distribution of TBL, both in GF newborn piglets before and after colonization with *Escherichia coli* and in older conventionally reared (CONV) piglets. The number of immunoglobulin (Ig)-secreting cells measured by the ELISPOT method was not influenced by microflora and food antigens. IgM-positive cells secreting IgM and CD45RC-positive cells spontaneously producing IgM, IgG, and IgA were detected in newborn thymus.

Our findings suggest that TBL differentiation and Ig switching to IgG and IgA-secreting cells is not influenced by external antigens and that the thymic microenviroment plays an important role in this process.


*Developmental Immunology*, 1998, Vol. 6, pp. 171–178
Reprints available directly from the publisher
Photocopying permitted by license only

© 1998 OPA (Overseas Publishers Association)
N.V. Published by license under the
Harwood Academic Publishers imprint,
part of the Gordon and Breach Publishing Group
Printed in Malaysia

### Antigenic Stimuli do not Influence Thymic B Lymphocytes: A Morphological and Functional Study in Germ-Free and Conventionally Reared Piglets

B. CUKROWSKA[a*] I. TREBICHAVSKÝ[a] P. ROSSMANN[a] Z. ŘEHÁKOVÁ[a] J. ŠINKORA[a] K. HAVERSON[b] R. LODINOVÁ-ŽÁDNÍKOVÁ[c] and H. TLASKALOVÁ-HOGENOVÁ[a]

[a]*Department of Immunology and Gnotobiology, Institute of Microbiology, Academy of Sciences of the Czech Republic, Vídeňská 1083, 142 20 Prague 4, Czech Republic*; [b]*Department of Clinical Veterinary Science, University of Bristol, Langford BS18 7DY, United Kingdom*; [c]*Institute for Care of Mother and Child, Prague, Czech Republic*

*(Received 12 August 1996; Accepted 14 August 1996; In final form 2 May 1997)*

We have recently reported that thymic B lymphocytes (TBL) are the first B-cell subpopulation undergoing isotype switching to IgG and IgA during embryonic life. The aim of this study is to analyze the influence of antigenic stimulation on TBL location and activity using a germ-free (GF) newborn pig model, in which maternal antibodies and antigens do not affect B-cell development. Immunohistological analysis showed that TBL were disseminated mainly in the thymic medulla. There were no differences in the distribution of TBL, both in GF newborn piglets before and after colonization with *Escherichia coli* and in older conventionally reared (CONV) piglets. The number of immunoglobulin (Ig)-secreting cells measured by the ELISPOT method was not influenced by microflora and food antigens. IgM-positive cells secreting IgM and CD45RC-positive cells spontaneously producing IgM, IgG, and IgA were detected in newborn thymus.

Our findings suggest that TBL differentiation and Ig switching to IgG and IgA-secreting cells is not influenced by external antigens and that the thymic microenviroment plays an important role in this process.

*Keywords:* Thymic B lymphocytes, B-cell differentiation, ELISPOT, pig

### INTRODUCTION

The existence of thymic B lymphocytes (TBL) as a constant component of the thymic medulla has been described recently (Isaacson et al., 1987; Inaba et al., 1988, Andreu-Sanchez et al., 1990; Bianchi et al., 1992; Spencer et al., 1992). Unique phenotypic and morphological features of TBL distinguish them from

*Corresponding author. Present address: Department of Immunology and Gnotobiology, Institute of Microbiology, Academy of Sciences of the Czech Republic, Vídeňská 1083, 142 20 Prague 4, Czech Republic.

peripheral lymphocytes. The majority of murine and human TBL express the CD5 molecule (Inaba et al., 1988; Punnonen and de Vries, 1993), but, in contrast to B-1 cells, TBL are derived from the bone marrow (Than et al., 1992). Human TBL also bear the CD2 antigen, characteristic for human T lymphocytes (Punnonen and de Vries, 1993). Recently, fetal TBL from human and pig have been shown to undergo isotype switching to IgA and IgG-secreting cells in a similar fashion as mature conventional B cells (Punnonen and de Vries, 1993; Cukrowska et al., 1996a). The mechanism of activation and the factors influencing TBL differentiation to immunoglobulin (Ig)-secreting cells remain unclear. The importance of direct T-cell interaction and cytokines in their maturation has been recently confirmed by functional analyses of murine and human TBL (Inaba et al., 1990, 1995; Punnonen and de Vries, 1993).

To investigate the characteristics of TBL, we used a unique pig model. In contrast to mice and humans, in which B-cell development is influenced by maternal antibodies and antigens (Kearney and Vakil, 1986), pigs have a special six-layered placenta preventing the transfer of maternal regulatory factors to the embryo. Transfer of maternal Igs occurs after suckling maternal colostrum (Šterzl et al., 1965). For this reason, pig fetuses and colostrum-deprived piglets reared in germ-free (GF) conditions are very useful experimental models that allow to differentiate between innate factors and factors arising under the influence of passively acquired antibodies and external antigenic stimuli (Tlaskalová-Hogenová et al., 1983). We have observed, using the ELISPOT method, that the fetal pig thymus contains Ig-secreting cells very early in embryonic life (Prokešová et al., 1981; Cukrowska et al., 1996a). In contrast to fetal splenic B cells, which have been found to produce exclusively IgM, fetal thymic B cells of the same age undergo spontaneous and after *in vitro* polyclonal activation isotype switching into IgG and IgA (Cukrowska et al., 1995, 1996a).

In the present study, we analyze the influence of external antigenic stimuli on spontaneous Ig production by TBL and on the location of B cells in the pig thymus.

### RESULTS

### Location of Thymic B Cells in GF Newborns, GF Piglets Colonized with *Escherichia coli* and Conventionally Reared (CONV) Piglets

The newborn pig thymus displayed sparsely scattered lymphocytes expressing membrane-bound Igs (Fig. 1, left). Most cells were seen in the medulla, without an apparent accumulation or follicular structures. There was no agglomeration around the Hassall's bodies. Single contoured cells were noted in the cortex in lower numbers than in the medulla (by 1 to 2 orders of magnitude). The reaction with the pig anti-rabbit antibody was negative. The deep cortex and medulla harbored larger cells with coarse cytoplasmic DAB-reactive granules (macrophages). This staining of macrophages was unchanged after omission of the antibodies (Fig. 1, right).

The adult pig thymus exhibited a similar distribution of Ig-positive lymphocytes as in GF newborn piglets; macrophages were concentrated in the corticomedullary junction (data not shown). The stimulation of GF piglets by *Escherichia coli O86* elicited an increase in endogenous peroxidase-reactive macrophages, whereas Ig-positive lymphocytes remained scarce and no follicular agglomeration was found (Fig. 2, left). In contrast to the scattered distribution of TBL, splenic Ig-positive cells were assembled to follicles (Fig. 2, right).

### Spontaneous Ig Production Measured by the ELISPOT Assay

As shown by immunohistological studies, the location and organization of TBL was not altered by antigenic stimuli; we also tried to analyze the functional state of these cells by measuring spontaneous Ig production. Conventional microflora and food antigens influenced neither the number of spot-forming cells nor the isotype pattern of Ig production by TBL. In contrast to splenic B cells of CONV piglets that showed a significant increase in Ig-secreting cells of all isotopes, the thymus of CONV piglets and GF newborns contained comparable numbers of IgM-, IgG-, and IgA-secreting cells (Figure 3). The experimental

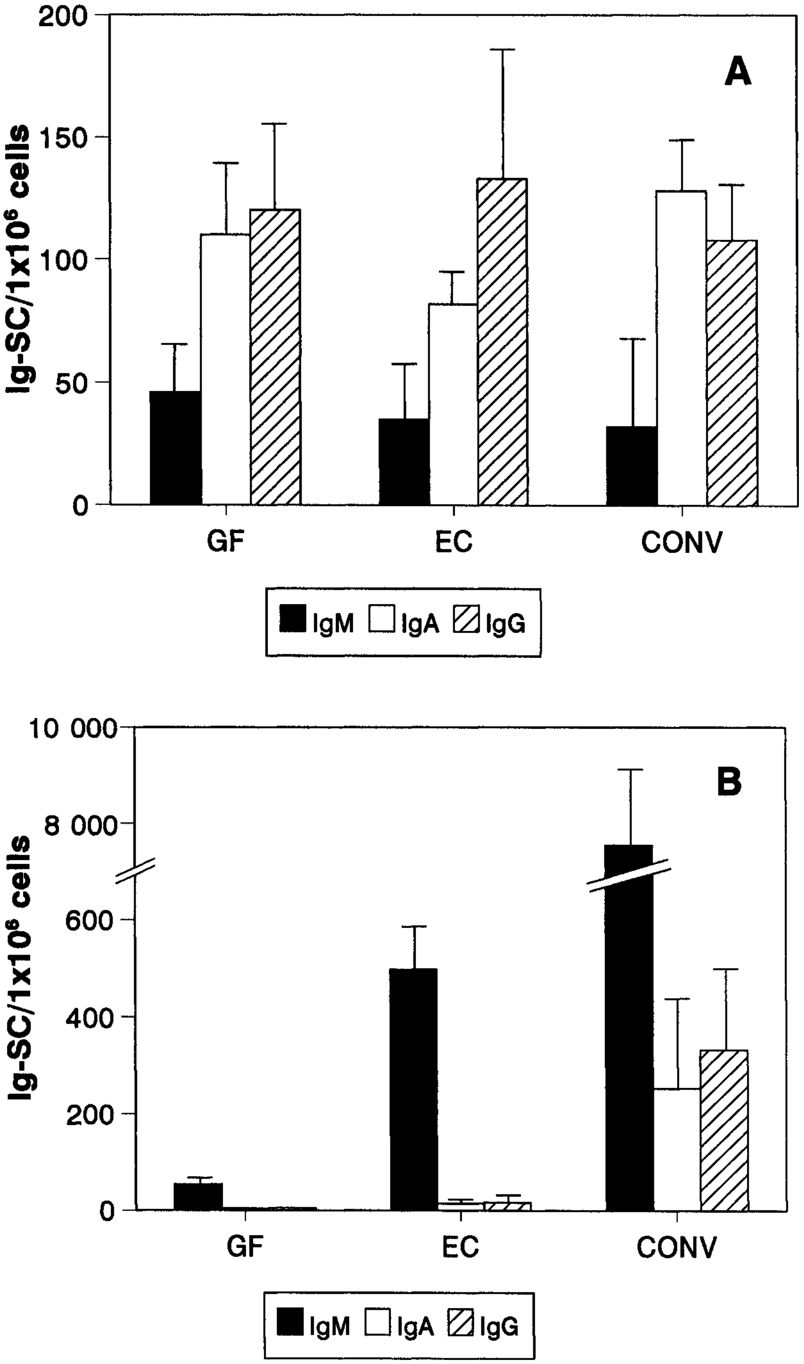

FIGURE 3 Spontaneous Ig production in (A) thymus and (B) spleen of GF newborns, GF piglets colonized by *E. coli O86* and CONV piglets. Ig production in thymic- and splenic-cell suspensions was measured by the ELISPOT method. The results are determined as the number of Ig-secreting cells (Ig-SC) per $10^6$ nucleated cells; arithmetical means ± SD from four animals are shown. GF, GF newborns; EC, GF piglets colonized by *E. coli*; CONV, conventionally reared piglets.

role in immune response against microflora, the problem of their biological functions is still open. The role of TBL as antigen-presenting cells in shaping of T-cell repertoire has been proposed (Inaba et al., 1991, Mazda et al., 1991). Mouse thymic B cells, but not splenic cells were found to induce tolerance by a deletion mechanism. However, in the light of recent findings on the importance of natural autoantibodies in selection processes (Holmberg and Kearney 1994), it cannot be excluded that Igs spontaneously secreted by TBL locally influence T-cell development. Analyses of the antibody specificity produced by TBL during ontogeny is still lacking. Interestingly, we have recently reported a pronounced reactivity of "preimmune" Igs, that is, isolated from fetal and GF newborn sera with pig thymocytes (Tlaskalová-Hogenová et al., 1994; Cukrowska et al., 1996b).

### MATERIALS AND METHODS

### Animals

Colostrum-deprived Miniature Minnesota pig newborns 1 day old were used. Some fetuses were kept in isolators after a gnotobiotic hysterectomy and were given a sterilized milktype diet (Travníček et al., 1966). The germ-free state was controlled twice a week by an aerobic and anaerobic cultivation of a

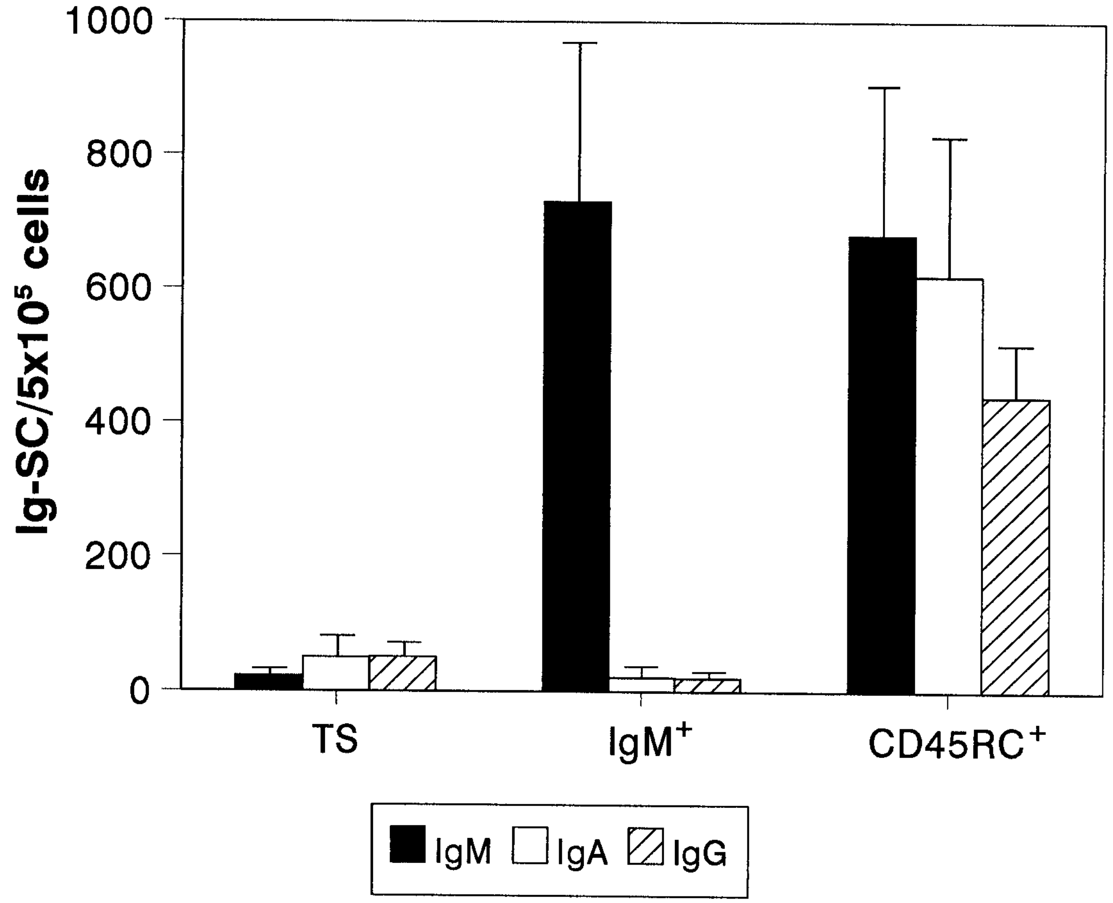

FIGURE 4 Spontaneous Ig production by thymic IgM-positive and CD45RC-positive cells. Cells were separated from GF newborn thymocytes on miniMACS column by anti-pig IgM and anti-pig CD45RC mAb. Ig production was measured by the ELISPOT method. The results are determined as the number of Ig-secreting cells (Ig-SC) per $5 \times 10^5$ nucleated cells; arithmetical means ±SD from five animals are shown. TS, thymic-cell suspension.

rectal swab. GF piglets received $5 \times 10^8$ non-pathogenic *E. coli O86* in the milk from the fifth day after the birth. On day 7 after colonization, animals were exsanguinated under anesthesia and lymphoid organs were taken for cell isolation. CONV piglets, 2 to 3 months old were also used.

### Isolation of Cells

The lymphoid organs were gently minced in RPMI-1640 medium, filtrated trough a nylon mesh, washed three times in the medium, and than resuspended in the complete RPMI-1640 medium supplemented by 10% fetal calf serum and gentamycin.

### Immunohistochemistry

Pig Ig-positive cells were detected on acetone-extracted cryostat sections (6 $\mu$m) of unfixed frozen pig thymus by one-step direct immunoenzyme test using the HRP-labeled rabbit anti-swine antibody (RASW-HRP) diluted 1:40 (Sevac, Prague, Czech Republic). The incubation was 1 hr at room temperature and the bound enzyme was developed with DAB-$H_2O_2$ reagent. The control test involved incubation with an antibody of different specificity (HRP-coupled swine anti-rabbit) or incubation with DAB-$H_2O_2$ alone.

### Detection of Spontaneously Ig-Secreting Cells

The number of spontaneously Ig-secreting cells of all isotypes was determined by the ELISPOT method using anti-pig isotype-specific mAb as previously described (Cukrowska et al., 1996a). The following murine anti-pig mAb were used: anti-IgM (LIG-4) (Dvořak et al. 1986), anti-IgG (23.3 1a), anti-IgA (27.9.1), anti-light chain (27.7.1 and 27.2.1) (van Zaane and Holst, 1987) (the kind gift from A.T.J. Bianchi, Lelystad, the Netherlands). In order to confirm that spot formation was constituted as a result of Ig synthesis during the *in vitro* incubation, an inhibition of protein synthesis by cycloheximide (Sigma, St.Louis) was conducted (Cukrowska et al., 1996a). This treatment abrogated the spot formation of all isotypes, thus demonstrating that this assay detects cells synthesizing and actively secreting Igs.

### Enrichment of B Lymphocytes from Thymus

A positive separation procedure by the magnetic miniMACS column (Miltenyi Biotech, Bergish-Gladbach, Germany) was performed using anti-pig IgM and anti-pig CD45RC mAb (Saalmüller 1996) to enrich B lymphocytes from newborn thymus according to manufactural instructions. Separated lymphocytes were spun down, resuspended in 10% RPMI-1640 medium, and used for detection of Ig secretion by the ELISPOT method. The purity of separated cells was >90% as assessed by flow cytometry. Magnetic microbeads and/or mAb had no effect on spontaneous Ig secretion (data not shown); thus, we could rule out the possible activation of cells by mAb and magnetic microbeads.

### *Acknowledgements*

The authors thank Andre Bianchi for the generous gift of the isotype-specific mAbs. This work was supported by grants 303/96/1256, 302/94/0051 from the Grant Agency of the Czech Republic, grant 3761-3 from the Grant Agency of the Ministry of Health of the Czech Republic and EC copernicus grant CIPA-CT-940209.

### *References*

Andreu-Sanchez J.L., Faro J., Alonso J.M., Paige Ch. J., Martinez A.C., and Marcos M. (1990). Ontogenic characterization of thymic B lymphocytes. Analysis in different mouse strains. Eur. J. Immunol. **20**: 1767-1773.

Bianchi A.T.J., Zwart R.J., Jeurissen S.H.M., and Moonen-Leusen H.W.M. (1992). Development of the B- and T-cell compartments in porcine lymphoid organs from birth to adult life: An immunohistological approach. Vet. Immunol. Immunopathol. **33**: 201-221.

Cukrowska B., Šinkora J., Mandel L., Šplíchal I., Bianchi A.T.J., Kovářů F., and Tlaskalová-Hogenová H. (1996a). Thymic B cells spontaneously produce IgM, IgG and IgA in pig fetuses and germ-free pigs: Detection by ELISPOT method. Immunology **87**: 487-492.

Cukrowska B., Šinkora J., Řeháková Z., Šinkora M., Šlíchal I., Tučková L., Avrameas S., Saalmüller A., Barot-Ciorbaru R., and

Tlaskalová-Hogenová H. (1996b). Isotype and antibody specificity of spontaneously formed immunoglobulins in pig fetuses and germ-free piglets: Production by CD5$^-$ B cells. Immunology **88**: 611-617.

Cukrowska B., Šinkora J., Řeháková Z., Šplíchal I., Tučková L., Barot-Ciorbaru R., Bianchi A.T.J., Lodinová-Žadniková R., and Tlaskalová-Hogenová H. (1995). Polyclonal immunoglobulin response of thymic, hepatic and splenic lymphocytes from fetal, germ-free and conventionally reared pigs to different B cell activators. Folia Microbiol. **40**: 421-430.

Dvořák P., Dvořákova D., Hruban V., Hořak V., and Staněk R. (1986). Monoclonal antibodies specific for pig serum and cell surface IgM. Res. Vet. Sci. **41**: 356-361.

Fend F., Nachbaur D., Oberwasserlechner F., Kreczy A., Huber H., and Müller-Hermelink H.K. (1991). Phenotype and topography of human thymic B cells. An immunohistologic study. Virchows Archiv. B Cell.Pathol. **60**: 381-388.

Holmberg D., and Kearney J. (1994). Selection of the B-cell repertoire and natural autoantibodies. In Autoimmunity: Physiology and Disease, Coutinho A., and Kazatchkine M.D., Eds. (New York: Wiley-Liss), pp. 89-107.

Inaba M., Inaba K., Adachi Y., Nango K., Ogata H., Muramatsu S., and Ikehara S. (1990). Functional analyses of thymic CD5$^+$ B cells. Responsiveness to Major Histocompatibility Complex class II-restricted T blasts but not lipopolysaccharide or anti-IgM plus interleukin 4. J. Exp. Med. **171**: 321-326.

Inaba M., Inaba K., Fuluba Y., Mori S., Haruna H., Doi H., Adachi Y., Iwai H., Hosaka N., and Hisha H. (1995). Activation of thymic B cells by signal of CD40 molecules plus interleukin-10. Eur. J. Immunol. **25**: 1244-1248.

Inaba M., Inaba K., Hosono M., Kumamoto T., Ishida T., Muramatsu S., Masuda T., and Ikehara S. (1991). Distinct mechanisms of neonatal tolerance induced by dendritic cells and thymic B cells. J. Exp. Med. **173**: 549-559.

Inaba M., Kuma S., Inaba K., Ogata H., Iwai H., Yasumizu R., Muramatsu S., Steiman R.M., and Ikehara S. (1988). Unusual phenotype of B cells in the thymus of normal mice. J. Exp. Med. **168**: 811-816.

Isaacson P.G., Norton A.J., and Addis B.J. (1987). The human thymus contains a novel population of B lymphocytes. Lancet **2**: 1488-1491.

Kearney J., and Vakil M. (1986). Idiotype-directed interactions during ontogeny play a major role in the establishment of the adult B cell repertoire. Immunol. Rev. **94**: 39-50.

Mazda O., Watanabe Y., Gyotoku J.I., and Katsura Y. (1991). Requirement of dendritic cells and B cells in clonal deletion of MLs-reactive T cells in the thymus. J. Exp. Med. **173**: 539-547.

Prokešová L., Trebichavský I., Kovářů F., Kostka J., and Rejnek J. (1981). Ontogeny of immunoglobulin synthesis. Production of IgM, IgG and IgA in pig foetuses. Dev. Comp. Immunol. **5**: 491-499.

Punnonn J., and de Vries J.E. (1993). Characterization of a novel CD2$^+$ human thymic B cell subset. J. Immunol. **151**: 100-110.

Saalmüller A. (1996). Characterization of swine leukocyte differentiation antigens. Immunol. Today **17**: 352-354.

Spencer J., Choy M., Hussell T., Papadaki J., and Isaacson P.G. (1992). Properties of human thymic B cells. Immunology **75**: 596-600.

Šterzl J., Rejnek J., and Travníček J. (1965). Impermeability of pig placenta for antibodies. Folia Microbiol. **11**: 7-10.

Than S., Inaba M., Inaba K., Fukuba Y., Adachi Y., and Ikehara S. (1992). Origin of thymic and peritoneal Ly-1 B cells. Eur. J. Immunol. **22**: 1299-1303.

Tlaskalová-Hogenová H., Mandel L., Trebichavský I., Kovářů F., Barot R., and Šterzl J. (1994). Development of immune responses in early pig ontogeny. Vet. Immunol. Immunopathol. **43**: 135-142.

Tlaskalová-Hogenová H., Šterzl J., Štěpáková R., Dlabač V., Větvička V., Rossmann P., Mandel L., and Rejnek J. (1983). Development of immunological capacity under germfree and conventional conditions. Ann. N.Y. Acad. Sci. **409**: 96-113.

Travníček J., Mandel L., Lanc A., and Růžička R. (1966). Rearing of germfree piglets (in Czech). Čs. Fysiol. **15**: 240-246.

Van Zaane D., and Hulst M.M. (1987). Monoclonal antibodies against porcine immunoglobulin isotypes. Vet. Immunol. Immunopathol. **16**: 23-36.

